# Imaging Characteristics of Breast Cancer in Women Aged 80 Years and Older: A Single-Center Experience

**DOI:** 10.5152/eurasianjmed.2026.261379

**Published:** 2026-06-12

**Authors:** Ayça Seyfettin, Serap Gültekin, Mecit Kantarcı

**Affiliations:** 1 Department of Radiology, Özel Ankara Güven Hospital, Ankara, Türkiye; 2 Department of Radiology, Gazi University Faculty of Medicine, Ankara, Türkiye; 3 Department of Radiology, Atatürk University Faculty of Medicine, Erzurum, Türkiye

**Keywords:** BI-RADS, breast cancer, elderly patients, mammography, radiologic findings, screening

## Abstract

**Background:**

Breast cancer in very elderly women presents unique clinical and imaging characteristics, and data regarding this age group remain limited. This study aimed to evaluate the imaging characteristics of breast cancer in patients aged 80 years and older together with clinical and pathological findings.

**Methods:**

A retrospective analysis was performed on 23 patients aged 80 years and older diagnosed with breast cancer at a single institution between January 2021 and January 2026. Clinical presentation, tumor size, histological grade, metastasis status, and imaging findings, including mammographic features and Breast Imaging Reporting and Data System (BI-RADS) categories, were evaluated. Associations between clinical presentation and tumor characteristics were statistically analyzed.

**Results:**

Of the patients, 30.4% were diagnosed during screening and 69.6% following diagnostic presentation. Tumor size was significantly larger in the diagnostic group than in the screening group (median: 24.5 mm vs. 14.5 mm; *P* < .001). Distant metastasis occurred only in diagnostically presenting patients (31.3%), while lymph node metastasis rates were similar between groups (*P* > .05). Grade 2 tumors were the most common (39.1%). The most frequent mammographic findings were masses with or without calcifications, most commonly irregular in shape, with spiculated margins and high density. BI-RADS 5 was the most frequent category (60.9%).

**Conclusion:**

Breast cancer imaging features in women aged 80 years and older resemble those of the general population. Smaller tumor sizes and absence of distant metastasis in screen-detected cases suggest that screening may still support earlier detection in selected very elderly women.

**Main Points**

- Breast cancer in women aged 80 years and older demonstrates imaging characteristics that are largely similar to those observed in the general population.
- Tumors detected through screening were significantly smaller than those diagnosed after symptomatic presentation.
- Distant metastasis was observed only in patients presenting diagnostically, while no metastatic disease was detected in screen-detected cases.
- Selected very elderly women may still benefit from breast cancer screening when individualized clinical considerations are applied.

## Introduction

Breast cancer is the most common cancer among women worldwide, affecting millions each year. According to the World Health Organization, approximately 2.3 million women were diagnosed with breast cancer globally in 2022, resulting in over 685 000 deaths.^1^ Notably, 71% of these cases occurred in women aged 50 years and older.

In the United States, as of 2024, 11% of invasive breast cancers and 5% of ductal carcinoma in situ (DCIS) cases were diagnosed in women aged 80 years and older.^2^Although specific national data are lacking for this country, breast cancer incidence rates in women aged 80 and above are significant.^3^

The unique characteristics of this age group—including increased comorbidities, frailty, and reduced life expectancy—necessitate their evaluation as a distinct subgroup.^4^ Accordingly, several studies have investigated the clinicopathological features of breast cancer in patients aged 80 years and older.^5^^-^^8^

While there is research regarding the necessity of mammographic screening in this age group, studies specifically examining imaging characteristics in women aged 80 and above remain scarce.^9^^,^^10^ This study aimed to present the imaging features of breast cancer in women aged 80 years and older, supported by clinical and pathological data.

## Material and Methods

This retrospective, cross-sectional, single-center observational study included 23 patients aged 80 years and older who were diagnosed with breast cancer at Ankara Güven Hospital Breast Unit institution between January 2021 and January 2026. This study was approved by Ethics Committee of Ankara Ufuk University (approval no: 26.01.02.01/05; date: January 02, 2026).

Patient demographics, clinical presentation, tumor size, histological grade, lymph node involvement, and distant metastasis status at diagnosis were retrieved from the hospital’s electronic medical records. Patients were classified according to their mode of presentation as either screening-detected or diagnostic.

Distant metastasis and lymph node involvement were recorded as present or absent. Mammographic images were reviewed through the Picture Archiving and Communication System (PACS) system. Breast parenchymal density was categorized as Type A (almost entirely fatty), Type B (scattered fibroglandular densities), Type C (heterogeneously dense), or Type D (extremely dense).

Lesions were categorized as mass only, mass with calcifications, calcifications only, asymmetry, or architectural distortion. For masses, morphological features were assessed, including shape (irregular, round, oval), margin (spiculated, microlobulated, indistinct, obscured), and density (high density, equal density).^11^

Based on mammographic findings, supplemented by ultrasound and breast magnetic resonance imaging (MRI) when available, final BI-RADS categories were assigned.

Statistical analysis was performed using IBM SPSS Statistics for Windows, Version 26.0 (IBM Corp., Armonk, NY, USA). Sample size was feasibility-based (all consecutive eligible cases, Jan 2021-Jan 2026); no a priori power calculation was performed due to the descriptive, single-center design.

The Shapiro–Wilk test was used to assess normality of continuous variables. For non-normally distributed data, the Mann–Whitney *U*-test was applied to compare groups. Relationships between categorical variables were analyzed using the Pearson chi-square test or Fisher’s exact test, depending on cell frequencies. A *P*-value <.05 was considered statistically significant.

## Results

A total of 23 patients were included in the study, with a mean age of 85.1 ± 3.4 years (range: 80-91 years). Based on clinical presentation at diagnosis, 7 patients (30.4%) were diagnosed during screening and 16 patients (69.6%) following diagnostic presentation. Among the diagnostic group, 3 patients were identified through incidental detection of a breast lesion on Positron Emission Tomography – Computed Tomography (PET-CT) performed for another primary malignancy, while the remaining 13 patients presented with symptomatic findings such as a palpable mass, breast hardness, skin changes, or nipple retraction.

Tumor sizes ranged from 8 mm to 55 mm. The mean tumor size was 25.18 mm, and the median was 24.5 mm. When patients were stratified according to clinical presentation, the median tumor size was 14.5 mm in the screening group and 24.5 mm in the diagnostic group. Tumors were significantly larger in patients who presented diagnostically compared to those diagnosed through screening (Mann–Whitney *U*-test, *P* < .001) (Table 1).

Distant metastasis occurred only in the diagnostic group (n = 5, 31.3%); however, this difference was not statistically significant (Fisher’s exact test, *P* > .05). There was no statistically significant difference in lymph node metastasis between the groups (screening: n = 2, 28.6% vs. diagnostic: n = 8, 50.0%; Fisher’s exact test, *P* > .05). Although low-grade tumors were more frequently observed in the screening group, the difference was not statistically significant (chi-square test, *P* > .05).

Mammographic evaluation revealed that the most common breast density patterns were Type B (n = 9, 39.1%) and Type C (n = 9, 39.1%). The most common lesion type was mass only (n = 8, 34.8%), followed by mass with calcifications (n = 7, 30.4%). Regarding mass morphology, irregular shapes were most frequent (n = 10, 43.5%), followed by oval (n = 4, 17.4%) and round (n = 2, 8.7%) shapes. The most frequent margin features were spiculated (n = 7, 30.4%) and indistinct (n = 6, 26.1%). High-density masses predominated (n = 13, 56.5%). The final BI-RADS categories assigned based on mammographic, ultrasonographic, and MRI findings revealed that BI-RADS 5 was the most frequent category (n = 14, 60.9%) (Table 2).

Pathological evaluation showed that invasive ductal carcinoma (IDC) was the most common histological type (n = 16, 69.6%). Estrogen receptor positivity was detected in 18 patients (78.3%), progesterone receptor positivity in 16 patients (69.6%), and HER2 negativity in 17 patients (73.9%) (Table 3).

### Representative Cases

#### Case 1: Screen-Detected Ductal Carcinoma In Situ

Patient information: 84-year-old female, asymptomatic, detected during routine screening.

Clinical findings: no palpable mass or skin changes.

**Figure 1.**
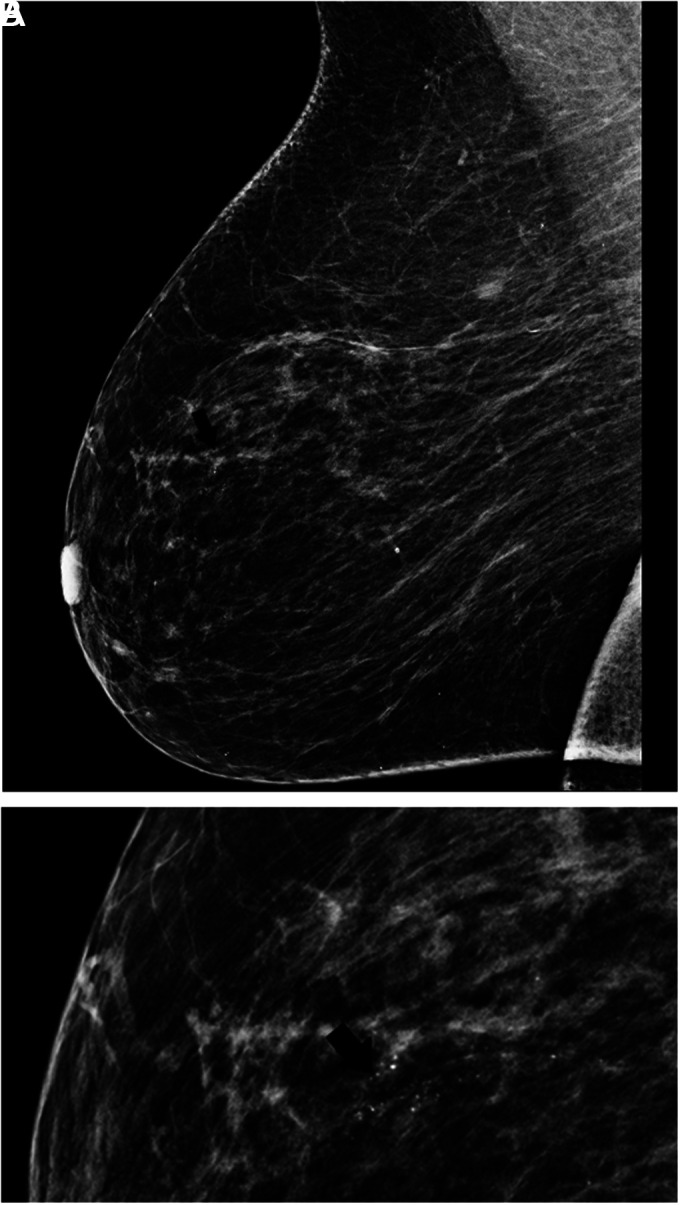
Mediolateral oblique mammographic image (A) of the case and magnified image of the calcifications (B).

Imaging findings:

- Mammography: segmentally distributed pleomorphic calcifications (arrow) in the upper outer quadrant of the right breast (Figure 1).
- Ultrasound: no corresponding mass.
- BI-RADS: Category 4C

#### Case 2: Symptomatically Detected Invasive Ductal Carcinoma

Patient information: an 81-year-old female with a palpable mass in the left breast.

Clinical findings: firm mass with nipple retraction.

**Figure 2.**
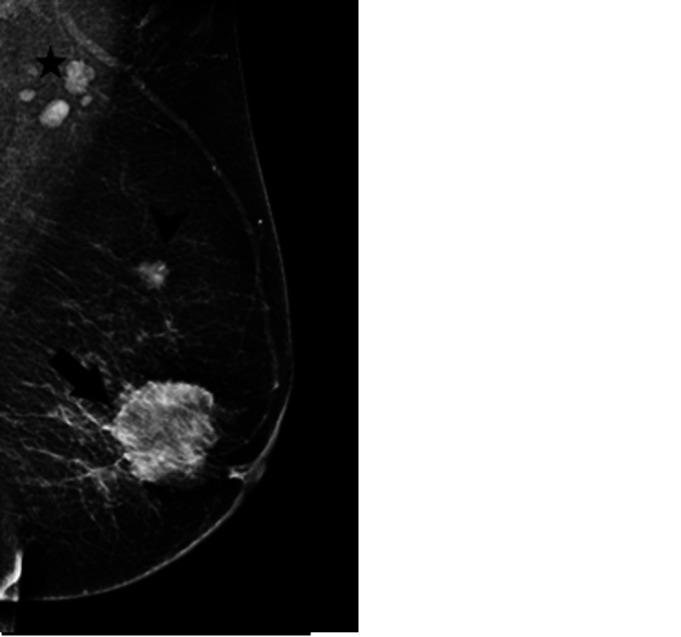
Craniocaudal (A) and mediolateral oblique (B) mammographic images of the case.

Imaging findings:

- Mammography: irregular high-density mass (arrow) with multicentric foci (arrow head) and axillary lymphadenopathy (star) (Figure 2).
- BI-RADS: Category 5.

#### Case 3: Screen-Detected Invasive Ductal Carcinoma

Patient information: 84-year-old female, asymptomatic, detected during routine screening.

Clinical findings: no breast symptoms.

**Figure 3.**
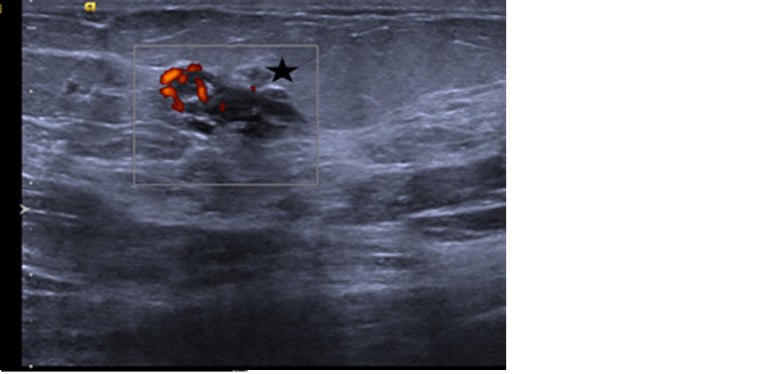
Right craniocaudal mammographic (A) and ultrasound images (B) of the case.

Imaging findings:

- Mammography: ovoid-shaped, microlobulated, high-density mass (arrow) (Figure 3).
- Ultrasound: hypoechoic, ovoid-shaped mass with microlobulated margins and internal vascularity (star) (Figure 3).
- BI-RADS: Category 4B

## Discussion

In the literature, it has been reported that more than 60% of breast cancers are detected at an asymptomatic stage through regular screening programs.^12^ In the study, however, only 27.3% of women aged 80 years and above were diagnosed during screening, while 72.7% were diagnosed following a diagnostic presentation. This rate is significantly higher than the diagnostic diagnosis rates reported in the general population (30%-35%). Screening participation is known to decrease substantially after the age of 75, and several recent studies have reported lower screening utilization rates among women aged 80 years and older compared with younger populations. Variability in guideline recommendations and the influence of comorbidities may partly explain this decline in screening uptake in very elderly women.^13^^,^^14^The findings highlight the low participation rates in screening programs among the elderly population and underscore the necessity of personalized screening strategies in this age group.

A significant difference in tumor size was observed according to the mode of diagnosis. The median tumor size was 14.5 mm in patients diagnosed via screening, compared to 28.0 mm in those diagnosed after a diagnostic presentation (*P* = .035). This result supports the contribution of screening programs to the detection of smaller tumors at earlier stages. Similarly, the literature reports that tumors detected through screening tend to be smaller and are associated with a better prognosis compared to those detected diagnostically.^15^Another recent study including women aged 75 years and older has similarly demonstrated that screen-detected tumors are significantly smaller at diagnosis and are more frequently identified at earlier stages compared with symptomatically detected cancers. These findings are consistent with the results and further support the potential role of individualized screening strategies in carefully selected very elderly patients.^16^

When metastatic status was evaluated, no distant organ metastases were observed in patients diagnosed through screening, whereas 25% of those diagnosed exhibited metastases. Although this difference did not reach statistical significance, it suggests a clinically meaningful trend. Recent studies evaluating elderly breast cancer populations have also demonstrated that screen-detected tumors are less frequently associated with advanced-stage disease or distant metastasis at the time of diagnosis.^5^^,^^13^^,^^17^

Regarding mammographic parenchymal density, Type B (40.9%) and Type C (40.9%) were the most frequently observed patterns in the cohort, followed by Type A (9.1%) and Type D (9.1%). Although breast density generally decreases with advancing age due to the progressive replacement of glandular tissue with fat, several studies have demonstrated that a considerable proportion of elderly women may still exhibit heterogeneously dense breast tissue at advanced ages. A study focusing on women aged 75 years and older reported a substantial presence of dense tissue even in the older age group, suggesting that age-related decline in density is not uniform across all individuals.^16^ Additionally, population-based research has shown that factors such as body mass index and age interact complexly with breast density patterns, and longitudinal analyses indicate variability in density changes over time across different age strata. These observations imply that persistent mammographic density could influence imaging characteristics and partially explain continued detection of breast cancer in very elderly populations, underscoring the importance of individualized radiologic evaluation rather than assumptions based solely on chronological age.^18^^-^^20^

When mammographic lesion types were evaluated, irregular and spiculated mass lesions were the most commonly observed findings in the study. This pattern is in keeping with the classical mammographic appearance of IDC, which has been consistently described as presenting with irregular shape, spiculated margins, and high radiographic density in multiple radiologic studies and BI-RADS-based analyses.^11^^,^^21^

Spiculated margins, in particular, are widely recognized as one of the most predictive mammographic features of malignancy, reflecting the desmoplastic reaction and infiltrative growth pattern typically associated with invasive carcinomas.^21^ In terms of lesion density, 68.2% of lesions in the study demonstrated high density, a characteristic frequently reported in malignant breast lesions compared with benign masses.^22^

These findings indicate that the mammographic characteristics observed in women aged 80 years and older in the study population largely parallel those reported in the general breast cancer population, suggesting that advanced age does not substantially alter the fundamental radiologic presentation of invasive disease.

In conclusion, breast cancer screening in women aged 80 years and above was associated with indicators of earlier-stage disease, including earlier diagnosis, smaller tumor sizes, and lower rates of distant metastasis. Enhancing screening participation and developing individualized approaches to support early diagnosis in the elderly population could significantly contribute to the fight against breast cancer.

### Study Limitations and Future Directions

The limitations of the study include the relatively small sample size and the single-center nature of the data. Future studies involving larger, multicenter cohorts are needed to validate these findings and to establish optimal screening strategies for the elderly population.

## Figures and Tables

**Table 1.** Demographic and Clinical Characteristics of the Patients

**Feature**	**Value**
Mean age (years)	85.1 ± 3.4 (80-91)
Presentation type	Screening: 7 (30.4%), diagnostic: 16 (69.6%)
Tumor size	Mean: 23.2 ± 8.2 mm, median: 24.5 mm (range: 14.5-55 mm)
Lymph node positivity	10 (43.5%)
Distant metastasis	5 (21.7%)

This table summarizes the age distribution, presentation type, tumor sizes, and metastasis status of patients aged 80 years and older included in the study.

**Table 2.** Mammographic and Imaging Findings

**Feature**	**Distribution**
Breast parenchymal density	Type A: 2 (8.7%), Type B: 9 (39.1%), Type C: 9 (39.1%), Type D: 3 (13.0%)
Lesion type	Mass: 8 (34.8%), mass + calcification: 7 (30.4%), calcification: 4 (17.4%), asymmetry: 2 (8.7%), distortion: 2 (8.7%)
Mass shape	Irregular: 10 (43.5%), oval: 4 (17.4%), round: 2 (8.7%)
Margin characteristics	Spiculated: 7 (30.4%), indistinct: 6 (26.1%), microlobulated: 5 (21.7%), obscured: 2 (8.7%)
Density	High: 13 (56.5%), iso-dense: 10 (43.5%)
BI-RADS category	BI-RADS 5: 14 (60.9%), 4C: 6 (26.1%), 4B: 3 (13.0%)

Findings from mammography, ultrasound, and breast magnetic resonance imaging are presented, including parenchymal density, lesion type, morphological features, and BI-RADS categories.

**Table 3.** Pathological and Molecular Characteristics

**Feature**	**Value**
Pathological type	IDC: 16 (69.6%), metaplastic: 2 (8.7%), ILC: 2 (8.7%), others: 3 (13.0%)
Tumor grade	Grade 1: 5 (21.7%), Grade 2: 9 (39.1%), Grade 3: 8 (34.8%), DCIS: 1 (4.3%)
ER positivity	18 (78.3%)
PR positivity	16 (69.6%)
HER2 negativity	17 (73.9%)

Histopathological results including tumor type, grade, and hormone receptor status are summarized.

DCIS, ductal carcinoma in situ; ER, estrogen receptor; IDC, invasive ductal carcinoma; PR, progesterone receptor; ILC, invasive lobular carcinoma.
